# Health Literacy and Mental Health in Adolescents: Parental and Self-Reported Cross-Sectional Data in a Bilingual Region

**DOI:** 10.3390/healthcare14131973

**Published:** 2026-07-02

**Authors:** Verena Barbieri, Dietmar Ausserhofer, Giuliano Piccoliori, Adolf Engl, Doris Hager von Strobele-Prainsack, Christian J. Wiedermann

**Affiliations:** Institute of General Practice and Public Health, Claudiana College of Health Professions, 39100 Bolzano, BZ, Italy; dietmar.ausserhofer@claudiana.bz.it (D.A.); giuliano.piccoliori@am-mg.claudiana.bz.it (G.P.); adolf.engl@gmail.com (A.E.); doris.hagerprainsack@am-mg.claudiana.bz.it (D.H.v.S.-P.); christian.wiedermann@am-mg.claudiana.bz.it (C.J.W.)

**Keywords:** adolescents, HLS-EU-Q16, HLSAC, mental health, psychosomatic complaints, parental perception, cross-cultural screening, German, Italian

## Abstract

**Highlights:**

**What are the main findings?**

- Adolescents completing the German questionnaire version reported higher HLSAC scores than those completing the Italian version; The association did not change after controlling for socioeconomic variables. Comparison of HLSAC on the item level showed better results for the German questionnaire version for items related to information seeking and appraisal as well as for self-awareness. Parental health literacy and parental completion rates relate to parental educational attainment, but not to questionnaire language.
- Lower parental and lower adolescents’ health literacy are related to higher adolescents’ mental health screening outcomes. Lower problematic Internet use and higher social support relate to higher parental and adolescent health literacy as well as to lower adolescent mental health screening outcomes.

**What are the implications of the main findings?**

- This cross-sectional study may provide information for further interventional healthcare planning. Enhancing health literacy through school-based programs may address parents, teachers, and adolescents. Future research needs detailed investigations on differences between adolescents completing German and Italian questionnaires. Research may focus on lower educated parents and adolescents having lower social support. Adolescents’ information seeking and information interpretation behavior needs detailed interventions.
- Longitudinal investigations may accompany the implementation process. The development of this bilingual process may be monitored. Existing German HL programs may be studied and—if meaningful—adapted to the bilingual context.

**Abstract:**

**Background/Objectives:** Improving the health literacy (HL) of adolescents and parents is an actual theme and needs detailed investigation in a bilingual region. **Methods:** In this cross-sectional school-recruited sample, parental and adolescent HL were associated with adolescent mental health screening outcomes. Exploratory indirect-effect models suggested possible pathways for future longitudinal research. About 3229 questionnaires provided information. Standardized internationally validated instruments were used. Parental and self-reported data were compared; the associations of HL with social support, problematic Internet use, questionnaire language, and mental health screening outcomes were explored. **Results:** Adolescents’ HL was associated with questionnaire language, with higher scores observed for German (23.1% high HL) than for Italian (14.3%). Higher levels of HL among both parents and adolescents were related to better mental health screening outcomes in adolescents with higher associations to adolescents’ HL. Social support and problematic Internet use were associated with both mental health screening outcomes and parental- and self-reported HL. HL related partly to the observed. Higher parental education was related to better parental HL and higher parental response rates. **Conclusions:** Enhancing HL among parents and adolescents through school-based programs needs language and education specific interventions. In the bilingual context of South Tyrol, the introduction of a bilingual program needs to be accompanied by scientific investigations, targeting language specific needs and differences. Existing international German school-based programs can be the basis for developing interventions to fit the Italian healthcare and educational system. South Tyrol offers the unique opportunity to introduce a scientific-based bilingual HL concept at schools. Enhancing information seeking and appraisal strategies in adolescents may be essential.

## 1. Introduction

In South Tyrol, a region in the north of Italy at the border to Austria and Switzerland, approximately 70% of the population speaks German and approximately 30% Italian. The healthcare system is managed according to the rules of the Italian-wide policies, but the information seek behavior is mainly embedded in the German context [1]. For the implementation of any regional bilingual intervention, it is important to understand the complex interplay of language, lifestyle, and social parameters in this bilingual region. An actual aim is to implement school-based HL programs on the regional level, involving teachers, parents, and adolescents.

A theoretical framework for the HL principle was developed during the last decades in the European context based on different definitions of HL. According to [2], HL is linked to literacy and entails people’s knowledge, motivation, and competences to access, understand, appraise, and apply health information in order to make judgments and make decisions in everyday life concerning healthcare, disease prevention, and health promotion to maintain or improve quality of life during the life course.

The European concept defining and measuring self-reported HL for adults [HLS-EU] [2,3] is available in the German [4,5] and Italian languages [6,7]. Findings in Italy suggest that inadequate HL is a prevailing problem. HL was found to be a mediator on the effect of socioeconomic status, associated with female gender, higher educational status, younger age, and not foreign nationality [8,9,10]. In Germany, a social gradient was observed [4], and actual longitudinal data of the Robert Koch Institute [11] show that after the COVID-19 pandemic, about 80% of the adult population in Germany had a low HL.

The HL of adolescents has been investigated in many countries through the HBSC studies [12]. Generally, slightly more students had a low HL in 2022 (24.4%) than in 2017/18 (21.4%) [13]. Differences in HL according to gender, age, type of school, and family affluence (FAS) were found. Low HL was associated with a high psychosomatic burden. Italian students [10] reported the lowest levels of HL compared with other countries. School connectedness and educational approach were the most relevant associated factors. Authors underscore the school’s role in reducing inequalities and promoting health for adolescents in Italy. Understanding the mechanisms and associates of HL is vital to finding adequate strategies in developing school-based HL programs [14].

In South Tyrol, a bilingual region, the question about HL assumes a new dimension. While it is known that there exist differences in HL measurement instruments between different countries [4,12], there is no existing evidence on the measured HL of different language groups within one healthcare system. When implementing a new HL intervention program in a bilingual region, the language aspect needs a good understanding of the actual situation.

In South Tyrol, four large repeated cross-sectional population-based screening surveys have been conducted since 2021 to monitor proxy- and self-reported mental health symptoms [15,16] among students aged 6 to 19 years. The studies provide data from a dual perspective, interviewing parents and adolescents. The surveys offered the possibility to screen for the HL of parents and adolescents in the region. The fourth wave of the survey included HLS-EU-Q16 and HLSAC instruments to monitor the actual HL levels. This approach provided a large cross-sectional dataset from school aged children and their parents. Additionally, it can give indications for the implementation of school-based HL programs and offers the possibility of linking HL screening data to mental health screening data.

Mental health screening was conducted using validated internationally established instruments. Such screening instruments are broad dimensional constructs, reflecting a population level approach. They cannot provide a diagnostic approach but can identify risk groups and can be related to DSM-5 [17] diagnostic groupings. Emotional, peer, conduct, and hyperactivity problems—thus internalizing and externalizing problems—were examined using the SDQ instrument. This instrument generally screens for mental health problems, while internalizing problems like depressive symptoms and anxiety symptoms were screened with more specific tools like the SCARED-GAD-9 and PHQ-2 instruments, respectively. General psychosomatic complaints were assessed using the HBSC-SCL instrument, capturing subjective health complaints. Large meta-analyses have shown that mental disorders as well as symptomatic mental health screening results increased significantly during the pandemic across countries and have not yet returned to pre-pandemic levels [18,19,20,21,22,23,24].

The link between HL and mental health screening data is important for intervention planning, as shown in several recent studies, because it can give an indication for future research. HL acts as a risk or protective factor, particularly in family and social contexts. It contributes not only to reduced symptoms but also to promotive mental health [25,26]. Better HL is linked to better mental health outcomes through several pathways. Mental health issues can be recognized earlier, behavior like lifestyle and coping strategies may be healthier, and better communication may have a positive influence on access to support. HL enhances the ability of emotional regulation, thus understanding the causes and trajectories and management options of stress [27], and facilitates symptom recognition and help seeking behavior [28]. Parental health literacy and children’s health outcomes are related [29], and in families with higher parental HL, communication tends to be more effective, supporting a shared understanding of health issues, collaborative decision-making, and engagement in preventive behaviors [30]. HL is related to efficient coping strategies and self-management [31], and is a core competence in information management when understanding, processing, and applying health information for decision-making [32,33].

Better health information helps mitigate adolescents’ psychosocial problems [34], and health illiteracy is a barrier in mental health treatment seeking [35]. An adequate HL serves as a protective factor for long-term mental health conditions [36] in adolescents. Detailed studies on both parental and adolescents’ HL literacy and adolescents’ mental health are rare. Recent studies show that adolescents’ well-being is associated with adolescents’ HL as well as with familiar social support [37]. Social support is a relevant aspect improving individuals’ HL, especially in young people. Therefore, social support should be addressed in interventions. HL can act as a mediator between social support and health outcomes [38].

Despite the broad HL framework, mental health issues may be related to the mental HL subdomain [39], focusing on the recognition, management, and prevention of mental disorders. It includes components such as symptom recognition, understanding of risk factors and treatments, and attitudes that facilitate appropriate help-seeking. It is conceptually centered on mental health specific behavior [40].

Another important and actually developing subdomain is digital HL. It represents a context-specific extension of HL, describing the competencies required to navigate and evaluate health information in digital environments [41]. It relates to the digital environment and is defined by the ability to seek, understand, critically evaluate, and apply health information from electronic sources to address health problems. Recent research shows that adolescents rely on online sources to supplement offline sources when seeking for health-related information. They address sensitive health questions and seek community, but they encounter challenges when assessing the credibility of online sources [42].

Further HL-related predictors are known from former studies. Low income and low educational status are linked to lower HL [8]. Despite social parameters, Internet addiction is related to lower critical HL [43], and more social media use is related to lower digital HL [44].

In the context of digital media, recent studies confirm that worse adolescent mental health outcomes are linked to elevated screen time and problematic social media use [45,46]. Additionally, lower digital health literacy significantly relates to problematic social media use [44,47]. In the 2022 German HBSC study, lower general HL in adolescents was associated with more problematic social media use [48]. Thus, the interaction of HL, mental health outcomes, and problematic media use needs specific attention.

This study focuses on general MH, providing a basis for future research that may include digital and mental HL for specific research questions. It even relates both adolescents’ HL and mental health outcomes to problematic Internet use in an exploratory way. Results may contribute to define more specific future research questions.

Pandemic-related research identified an association between mental health and HL [49] and emphasized the important and increasing role of school interventions. Thus, post-pandemic evidence about the relations between adolescents’ mental health and parental and self-reported adolescents’ HL is needed. The newest evidence suggests [50] to not only focus on individual HL in adolescents, but to consider the whole environment when implementing HL programs. Detailed investigations may provide indications for future school intervention planning in high income countries.

The first aim of the study was to assess the actual parental and adolescents’ HL and explore differences between the two questionnaire language groups. Understanding language specific and cultural differences may help to meet the special needs of the region when developing strategies to improve HL in the population. The bilingual setting of South Tyrol offers the unique opportunity to contribute to a broader understanding of self-reported HL using established questionnaires in both the German and Italian versions within one healthcare system. Second, the dual perspective provides the possibility of exploring how parental and adolescent HL are linked to each other and to other factors. These explorations may help develop targeted intervention programs for both parents and children throughout German and Italian school settings. Third, the exploration of the link between adolescents’ mental health screening results, the proxy- and self-reported HL, and associated factors may provide indications on the actual regional needs for the implementation of school-based HL programs, especially for vulnerable subgroups.

Recent research shows that adolescents’ and parental mental HL is linked to adolescents’ mental health with differences between the generations’ perspectives [25]. Our study offers the unique opportunity to investigate parental and adolescents’ HL using standardized and validated HL questionnaires in the German and Italian languages and to associate the results with mental health screening data of the same cohort in a post-pandemic sample.

The fourth cross-sectional COP-S (Corona and Psyche South Tyrol) study [15,16] relates the population-based post-pandemic mental health screening data of students to HL outcomes of parents and adolescents.

## 2. Materials and Methods

This population-based cross-sectional online survey was conducted between 17 March and 13 April 2025 using the SoSci Survey platform (Version 3.2.46, Munich, Germany). The survey was repeated for the fourth time after 2021, 2022, and 2023, and named Corona and Psyche South Tyrol (COP-S) [15,16]. It aimed to screen the mental health of children and adolescents in South Tyrol during and after the pandemic. The fourth wave of the study additionally collected comprehensive data on HL among children and adolescents. Recruitment was carried out through all provincial schools by contacting parents via email, sending a link to the anonymous questionnaire. No school or class clustering was performed due to privacy reasons. Parents of children and adolescents between 6 and 19 years were invited to complete a questionnaire. Additionally, adolescents between 11 and 19 years were invited to complete a self-report after their parents had completed the parent version. A reminder email followed after two weeks. Informed consent was obtained online from parents and adolescents prior to participation. More than 40,000 families were invited to participate.

The COP-S survey was designed following the German COPSY studies [23,51] and was adapted for the post-pandemic time and for South Tyrolean needs. According to the Italian school system, questionnaires were distributed to the parents of children aged between 6 and 19 years. Parent- and self-reports of adolescents between 11 and 19, going to lower and higher secondary school, were investigated in this study. Parents’ reports of children from elementary school (aged 6 to 10) were investigated separately [52].

Out of the 7818 available datasets from 6 to 19 years, 4496 regarded participants aged 11 to 19. Parent- and adolescent reports were analyzed and compared.

The study was conducted in accordance with the Declaration of Helsinki and approved by the Ethics Committee of the Autonomous Province of Bolzano, Italy (protocol code 11-2025, 19 February 2025). During the pandemic waves of the COP-S survey, support services, referral options, and emergency resources were provided at the end of the questionnaire. In the post-pandemic COP-S 4 wave, this information was distributed directly by schools. The online questionnaire was available in the German and Italian languages. In our survey, parents were asked for family language (i.e., the language mainly spoken at home). Participants could choose between German, Italian, Ladin, and other. Parents even stated the school language (in South Tyrol there exist German, Italian and Ladin schools). Associations between the three language types are presented in the Results section. Language specific analyses were conducted for questionnaire language.

### 2.1. Assessment of Parental and Adolescent HL

Self-assessed adolescent HL was measured using the HL for School-Aged Children (HLSAC) 10-item scale [10,53] with responses ranging from 1 = not at all true to 4 = absolutely true. The total sum score ranged between 10 and 40, with a lower score indicating lower HL. The scores were categorized as low (10–25), moderate (26–35), or high (36–40). Scores were calculated when all 10 questions were answered. The group of incomplete answers (1 to 9 answers available) was defined as the “missing” category and analyzed in the last subsection of the results.

Self-assessed parental HL was measured using the HLS-EU-Q16 questionnaire [5,7,54,55]. The total score ranged from 0 to 16, with higher scores indicating better HL with a Cronbach’s alpha of 0.799 for the Italian [7] and 0.88 for the German [5] version. Three levels of HL were defined as inadequate (0–8), problematic (9–12), and adequate (13–16). Scores were calculated when at least 14 out of the 16 questions were answered [7]. The group of incomplete answers (1 to 13 answers available) was defined as the “missing” category. The “missing” category is analyzed in the last subsection of the results.

**Figure 1:**
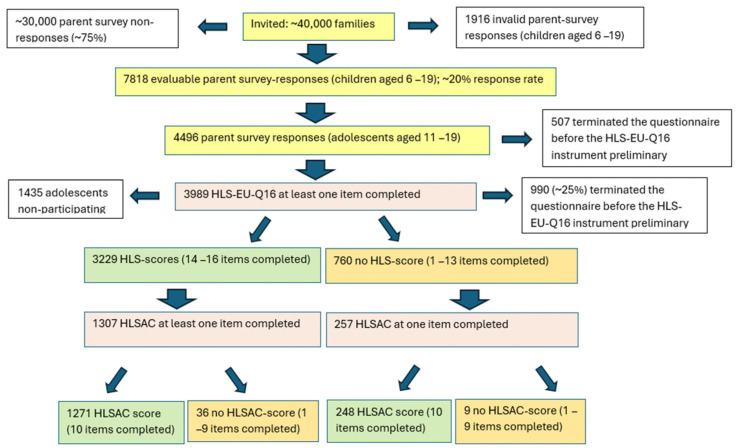
Datasets for the sum score calculations for HLS-EU-Q16 and HLSAC; yellow: total datasets in the survey; brown: datasets with at least partially completed HL questions; green: datasets with valid HL-scores (HLS-EU-Q16: 14–16 answers available; HLSAC: 10 answers available); dark yellow: datasets without valid HL scores (HLS-EU-Q16: 1–13 answers available; HLSAC: 1–19 answers available); Arrows indicate data selection process as described above.

Figure 1 illustrates how data distributed among the available and not available HL scores. Out of the available datasets, 3989 (88.7%) were at least partially completed regarding HLS-EU-Q16 score, while 507 (12.3%) participants aborted the survey without completing any HLS-EU-Q16 question. These 12.3% were not used for the analysis. A total of 760 (19.1%) cases were not appropriate to calculate the sum score (only 1–13 answers available). Out of the 3989 parent reports, 1564 self-reports of adolescents with at least partially completed HLSAC data were available, with 45 (2.9%) cases not appropriate for sum score calculations (only partially completed). Analyses were conducted using all datasets with an available sum score. We did not apply any weighting or input strategies. This decision was made at the beginning of our COP-S series.

### 2.2. Sociodemographic Variables and Problematic Internet Use

The sociodemographic variables included the age and gender of both parents and children, parental educational level assessed via the CASMIN index [56], single parenthood status, residency based on zip codes, and migration background.

Perceived social support was measured using the Multidimensional Scale of Perceived Social Support (MSPSS) [57] reported by parents and adolescents, and family socioeconomic status was measured with the Family Affluence Scale III (FAS III) [58,59,60,61] from the parent reports. The FAS III is a validated six-item measure of material assets and family living conditions like number of cars, bathrooms, own bedrooms, and family holidays.

Self-reported adolescent problematic Internet use was assessed using the German [62] and Italian [63] versions of the Generalized Problematic Internet Use Scale 2 (GPIUS-2) [64].

### 2.3. Mental Health Screening Measures

The Health Behavior in School-aged Children Symptom Checklist (HBSC-SCL) was employed to assess parent- and self-reported psychosomatic complaints. It identified eight psychosomatic problems during the past week: headache, stomachache, backache, feeling down, irritability, feeling nervous, sleep problems, and dizziness. Responses were recorded on a 5-point scale ranging from 1 = “daily” to 5 = “not at all” [65,66]. The number of different complaints per week was assessed as count data ranging from 0 to 8.

The parent- and self-reported SDQ (Strength and Difficulties Questionnaire) [67] assessed the mental well-being of adolescents across five dimensions: emotional symptoms, conduct problems, hyperactivity/inattention, peer relationship problems, and prosocial behavior. The total problem score was calculated from the first four subscales (excluding prosocial behavior) and ranged from 0 to 40, with higher scores indicating greater difficulties.

Self-reported Screen of Child Anxiety Related Emotional Disorders (SCARED): The Generalized Anxiety Disorder (GAD-9) subscale asked nine questions, such as “I worry about other people liking me”. It used a 3-point response scale (0, “not or hardly true” to 2, “very or often true”). The total score ranged from 0 to 18, with higher scores indicating more problems. Good validity and reliability of the GAD-9 subscale have been demonstrated in adolescent populations [68,69,70].

Self-reported Patient Health Questionnaire-2 (PHQ-2): The questionnaire asked two questions regarding depression on a 4-point Likert scale (from 0, “nearly never” to 3, “nearly every day”). It is recommended for adolescents aged 12 years and older and has been validated for the corresponding cultural background [71,72]. The total score ranged from 0 to 6, with higher values indicating more problems. In our study, the instrument was used for adolescents aged 11 years or older, according to [23,51].

### 2.4. Data Analysis

The primary outcome variables were HLS-EU-Q16 and HLSAC-scores. For all scores, reliability analysis per questionnaire language group was conducted using Cronbach’s alpha. Item level comparison between languages for HLSAC was conducted.

Descriptive statistics for continuous variables were calculated as means (M) with standard deviations (SD); nominal or categorical variables were presented as absolute counts and percentages. Group differences for two groups were tested using chi-square tests for nominal and categorical variables (effect size: Phi) and Mann–Whitney tests for non-normally distributed continuous variables (effect size according to [73] with small effect size ranging from 0.10–0.29; medium effect size from 0.30–0.49 and large effect size ≥ 0.5). The Kruskal–Wallis test was applied to compare three or more groups (effect size eta-squared with small effect size from 0.01–0.06; medium effect size: 0.06 < 0.14; large effect size ≥ 0.14). Pairwise post hoc tests were performed using the Bonferroni correction. Distributions of metric variables were visualized using Box plots.

Associations between nominal or ordinal variables were measured using the Phi coefficient; between dichotomous and continuous variables, we used point-biserial correlation; and for continuous variables, Spearman’s correlation coefficient was applied.

Jeffreys’ binomial confidence intervals were calculated for proportions. *p*-values < 0.001 are indicated with ***, <0.01 with **, <0.05, *, and *p*-values ≥ 0.05 are considered non-significant (n.s.). All statistical analyses were performed using SPSS version 27.0.0.0.

Linear regression analysis was calculated for the outcome HLS-EU-Q16 scores and HLSAC scores. A linear relationship between outcome and independent variables was assumed. Beta coefficients with 95% confidence intervals (CIs), standardized beta coefficients, and *p*-values were reported for each independent variable. Corrected R^2 was reported to account for model fit. Regression diagnostics checked for model assumptions of linear regression modeling, like independence and normality of residuals, homoscedasticity and multicollinearity (variance inflation factor VIF). Outliers were identified using centered leverage points.

One of our interests was to obtain indications of what may happen when establishing HL programs at schools with a special focus on young people having a lower MSPSS or higher digital media use. The hypothesis was that introducing HL programs at school may have an indirect effect on the already existing relation between MSPSS/GPIUS-2 and mental health outcomes [18].

An indirect effect model was implemented to explore the indirect effect of HLS-EU-Q16 and HLSAC on the association of the two different predictors MSPSS score and GPIUS-2 score with mental health screening outcomes. Due to the cross-sectional character of the data, the modeling approach was explorative. According to [74], we chose this approach, since in future, time dependent modeling approaches are planned. The aim is to introduce HL programs at school with a focus on vulnerable subgroups. Even if temporal ordering in a cross-sectional dataset is not given, we may obtain an idea on how HL may act in a future longitudinal dataset as an indirect effect. Our approach followed [75]. To avoid misinterpretation, we used “exploratory cross-sectional indirect effect modeling” instead of “mediation modeling”.

**Figure 2:**
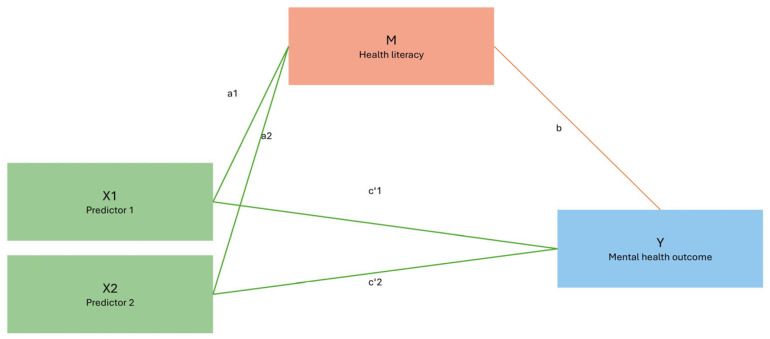
Indirect effect model 4 (SPSS PROCESS macro). The total effects c1 and c2 of the independent variables X1 and X2 with the outcome Y is modeled as c1 = c1′ + a1* b1 and c2 = c2′ + a2* b2, with c1′ the direct effect of the independent variable X1 and c2 the direct effect of the independent variable X2 on Y, a is the association of X with the indirect associate M, and b is the association of the indirect associate M with outcome Y.

Modeling was performed using the SPSS version 27.0.0.0 macro “PROCESS”, employing Model 4 with 5000 bootstrap samples. To check for robustness, the models were rerun with 10,000 bootstrap samples. The theoretical models are visualized in Figure 2.

#### Exploratory Cross-Sectional Indirect Effect Model Overview

Independent variables were selected based on the literature and significant associations with the dependent mental health variables. HLS-EU-Q16 score and HLSAC score were incorporated as indirect effects when they exhibited significant associations with both the independent variables and the outcome.

A indirect effect model was established based on the following requirements:

- Independent variables must show significant associations with the outcome;
- Independent variables must also show significant associations with the indirect effect;
- Both independent variables and indirect effect significantly predict the dependent variable in a regression model.

## 3. Results

Cronbach’s alpha for HLS-EU-Q16 was 0.928 for the German version and 0.928 for the Italian version. For HLSAC, it was 0.893 and 0.889, respectively. For SCARED-GAD-9, the values were 0.906 and 0.868 and 0.738 and 0.704 for PHQ-2. For proxy-reported SDQ, Cronbach’s alpha was 0.834 for the German and 0.845 for the Italian version, while for self-reported SDQ, the values were 0.817 and 0.843. For proxy-reported HBSC-SCL, Cronbach’s alpha was 0.771 for the German and 0.803 for the Italian version, while for self-reported HBSC-SCL, the values were 0.838 and 0.851, respectively. For GPIUS-2, the values were 0.927 and 0.928. For the German and Italian proxy-reported MSPSS, the Cronbach’s alpha was 0.985 and 0.970, respectively, and for self-reported MSPSS, the values were 0.979 and 0.960.

The results first describe parental and adolescent HL and general demographic characteristics of the dataset. Then, associations of parental and adolescents’ HL with questionnaire language and sociodemographic characteristics are examined. Finally, associations between parental and adolescents’ HL and mental health screening outcomes are explored.

Among the 4496 available parent reports of adolescents between 11 and 19 years, 3989 filled in at least one of the 16 HLS-EU-Q16 questions. Out of them, 7.3% had an inadequate, 18.8% a problematic, 54.8% an adequate HL, and 19.1% were incomplete and thus coded as missing. Of the 3229 complete cases, 9.0% had an inadequate, 23.2% a problematic HL, and 67.7% an adequate HL.

Of the 2030 adolescent self-reported cases, 1570 adolescents filled in at least one of the 10 HLSAC questions. Out of them, 10.7% had a low, 64.6% a middle, 21.8% a high HL, and 2.9% were incomplete and thus coded as missing. Of the 1525 complete cases, 11.0% had a low, 66.6% a medium, and 22.4% a high HL.

Parental HL was measured in 2649 (82.0%) German questionnaires and in 580 (18.0%) Italian questionnaires. Self-reported adolescents’ HL was reported in 1296 (85%) German and in 229 (15%) Italian questionnaires.

### 3.1. Demographic and Socioeconomic Characteristics of the Dataset

Among the 3229 parents, participants were predominantly (88.7%) female, and the mean age was 47.6 + −5.7 years. A total of 12.7% were single parents, 30.8% were urban residents, and 9.7% had a migration background. Regarding FAS III, 15.8% reported low socioeconomic status, 56.2% middle, and 28.0% high. The parent-reported MSPSS score was categorized low in 14.0%, moderate in 13.5%, and high in 72.6% of cases.

According to the CASMIN index, 16.8% of the parents had a low, 40.1% a middle, and 43.1% a high educational attainment For paired samples (if available), the educational level of the parent who completed the questionnaire was significantly higher than the educational level of the second parent who did not complete the questionnaire (Phi = 0.603; *p* < 0.001). Among the completing parents, 4.0% had a primary school degree, 21.7% a vocational school degree, 35.1% a high school degree, 37.0% a university degree, and 2.1% had a degree from abroad. In contrast, among the partners (not completing parent, if available), 10.3% had a primary school degree, 40.0% a vocational school degree, 25.6% a high school degree, 22.3% a university degree, and 1.7% had a degree from abroad.

According to the parent reports, 49.9% of the children were female, and the overall mean age was 14.5 + −2.4.

Among the 1525 self-reporting adolescents, the mean age was 14.4 + −2.3, 49.8% were female, 11.6% came from a single parent household, 28.8% were urban residents, 8.6% had migration background, 6.8% stated a low, 10.9% a moderate, and 82.3% a high self-reported MSPSS, and 17.4% had a low, 56.6% a middle, and 26.0% a high FAS III. Regarding parental education, 18.9% had a parent with low, 42.0% with medium, and 39.0% with a high CASMIN index.

The following demographic variables differed for German and Italian survey language: urban residency (Ger: 21.9 vs. Ita: 71.6%; Phi = 0.413; *p* < 0.001), migration background (Ger: 8.7% vs. Ita: 14.5%; Phi = 0.075; *p* < 0.001), CASMIN (Ger: 18.1% low; 41.0% medium; 40.9% high vs. Ita: 10.8% low; 36.2% medium; 53.0% high; Phi = 0.103; *p* < 0.001), FAS III (Ger: 14.8% low; 57.3% middle; 27.9% high vs. Ita: 20.6% low; 51.3% medium; 28.1% high; PHI = 0.064; *p* = 0.001), parental MSPSS (Ger: 15.2% low; 12.6% moderate; 72.3% high vs. Ita: 8.3% low; 17.8% moderate; 73.9% high; Phi = 0.089; *p* < 0.001), adolescents’ gender (Ger: 51.5% males vs. Ita: 45.3% males; Phi = 0.044; *p* = 0.012), and single parenthood (Ger: 11.7% vs. Ita: 17.5%; Phi = 0.067; *p* < 0.001). No difference was found for self-reported MSPSS categories.

### 3.2. HL Categories for German- and Italian-Completing Parents and Adolescents

While 82.0% of the participants filled in the German and 18% the Italian version of the questionnaire, approximately 83.6% declared that their child went to a German school, 13.2% went to an Italian school, and 2.6% chose the Ladin school. Furthermore, 78.2% declared speaking mainly German at home, 17.1% spoke Italian, 2.9% spoke Ladin, and 1.5% spoke another language. The relations between languages were high: Phi was 0.855 for questionnaire language and family language, and 0.768 for questionnaire language and school language.

**Figure 3:**
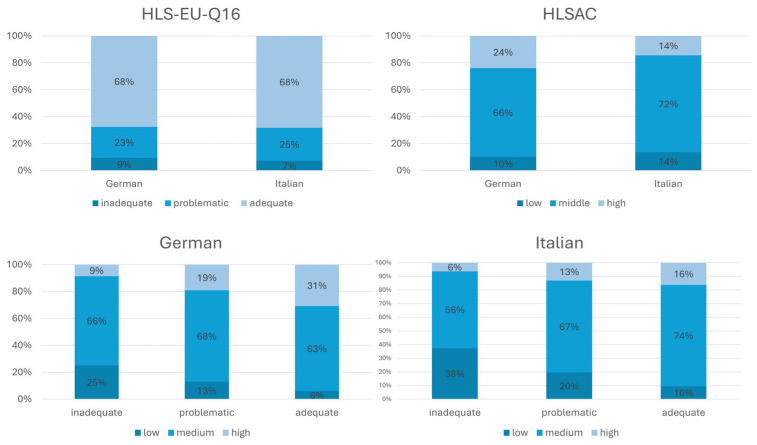
(**Upper panel**) HL categories for parents (HLS-EU-Q16) and adolescents (HLSAC) per the German and Italian questionnaires. (**Lower panel**) Adolescents’ HL (HLSAC; *y*-axis) categories per parental HL (HLS-EU-Q16; *x*-axis) categories for the German and Italian questionnaires.

Analyses of categorized HL are presented in Figure 3. There were 2649 (82.0%) German and 580 (18.0%) Italian questionnaires available. A total of 9.4% [8.4%; 10.6%] of the participants completing the German version resulted in an inadequate, 23.0% [21.4%; 24.6%] a problematic, and 67.6% [65.8%; 69.4%] an adequate HL, while 7.2% [5.3%; 9.6%] of the participants completing the Italian version had an inadequate, 24.5% [21.1%; 28.1%] a problematic, and 68.3% [64.4%; 72.0%] an adequate HL. There was no significant difference between questionnaire languages. For adolescents completing the German version, 10.6% [9.0%; 12.3%] reported a low, 65.7% [63.0%; 68.2%] a middle, and 23.8% [21.5%; 26.1%] a high HL. Adolescents completing the Italian questionnaire version reported low HL in 13.5% [9.6%; 18.4%] of the cases, 71.6% [65.5%; 77.2%] reported middle HL, and 14.8% [10.7%; 19.9%] high HL. There was a significant difference between questionnaire languages (effect size = 0.091; *p* = 0.009).

Cramer’s V for parent- and self-reported HL categories was 0.159 (*p* < 0.001) for the German questionnaires and 0.166 (*p* = 0.021) for Italian questionnaires. Figure 3 (lower panel) shows the distribution of adolescents’ HL categories among the parental HL categories.

### 3.3. HLS-EU-Q16 and HLSAC Scores and Associations to Questionnaire Language and to Demographic Variables

The HLS-EU-Q16 score did not significantly differ between the German (13.17 + −3.12) and Italian (13.3 + −2.94) questionnaire language while the HLSAC score differed significantly between the German (32.01 + −5.11) and Italian (30.61 + −4.80) questionnaire language (effect size = 0.091; *p* < 0.001). Parental and adolescent HL scores correlated positively for both questionnaire languages (German: 0.238; *p* < 0.001; Italian: 0.189; *p* < 0.001) according to Spearman’s correlation coefficient.

Due to the significant differences between the German and Italian questionnaire versions regarding HLSAC scores, we provided an item-level descriptive comparison between questionnaire languages in Table 1. Item level descriptions refer to [76]. Items 1 and 2 represent theoretical health knowledge and did not differ between language groups. Practical knowledge of item 3 regarding information seeking was significantly higher in the German questionnaires, while practical knowledge of item 4 regarding following medical doctors’ instructions did not differ significantly. Critical thinking regarding health-related information of items 5 and 6 was higher in the German questionnaires. Self-awareness of items 7 and 8 regarding one’s own health-related choices and behaviors was significantly higher in the German questionnaires. Citizenship of item 9 regarding their own actions in the natural environment did not differ between questionnaire languages, while having an idea on how to improve the immediate surroundings in item 10 was significantly higher for German questionnaire language.

**Figure 4:**
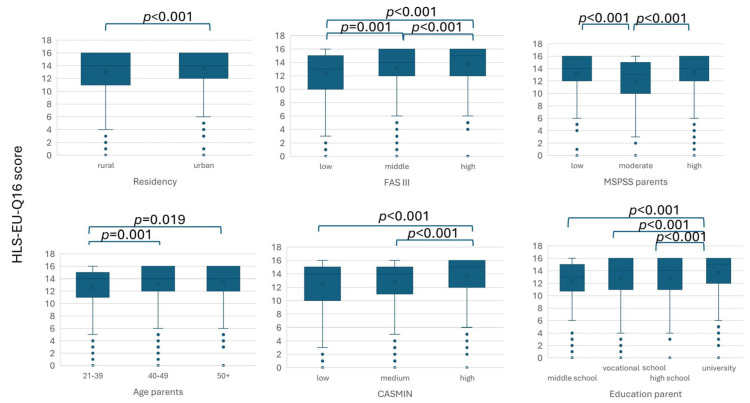
HLS-EU-Q16 score and its associations with demographic variables, *p*-values refer to Mann–Whitney tests for two groups and Bonferroni corrected post hoc tests of the Kruskal–Wallis test for more than two groups.

**Figure 5:**
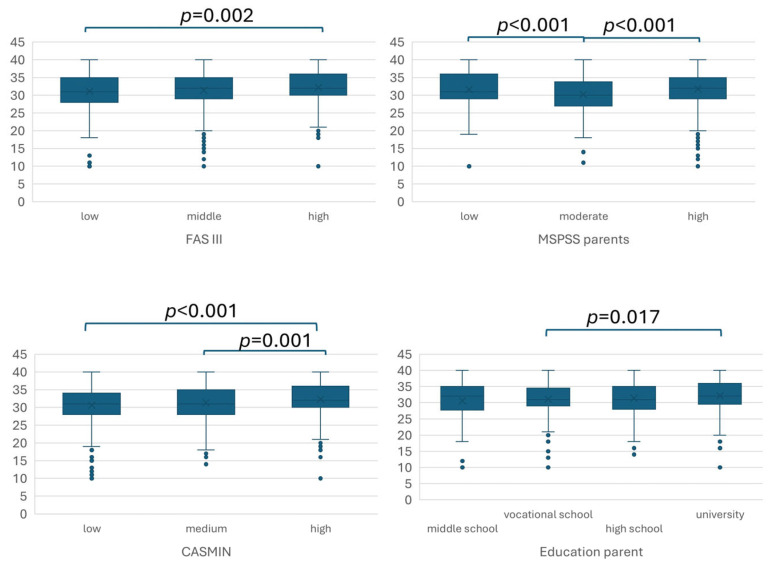
HLSAC score and its associations with demographic variables, *p*-values refer to Mann–Whitney tests for two groups and Bonferroni corrected post hoc tests of the Kruskal–Wallis test for more than two groups.

Figure 4 shows the HLS-EU-Q16 scores with Bonferroni corrected significant post hoc differences for demographic factors. Figure 5 shows the same for the HLSAC scores. Urban residents had a significantly higher HLS-EU-Q16 score than rural residents (effect size = 0.081; *p* < 0.001), and single parents had significantly lower HLS-EU-Q16 scores than not single parents (effect size = 0.035; *p* < 0.045).

HLS-EU-Q-16 differed significantly between parental age groups (effect size = 0.004; *p* < 0.001), and HLS-EU-16 and HLSAC differed significantly between MSPSS categories (effect sizes = 0.019 and 0.001; resp; *p* < 0.001, both) with the lowest values in the moderate group, and FAS III categories (HLS-EU-Q16: effect size = 0.016; *p* < 0.001; HLSAC: effect size = 0.004; *p* = 0.016) with higher scores in higher socioeconomic classes. HLS-EU-Q16 (effect size = 0.023; *p* < 0.001) and HLSAC (effect size = 0.013; *p* < 0.001) differed significantly between CASMIN index categories with higher scores in the highest educated group. HLS-EU-Q16 was not associated with parent’s gender and migration background. HLSAC was not associated with adolescents’ gender, migration background, single parenthood, and residency.

Since CASMIN index and parental education of the parent who filled in the questionnaire were highly correlated (0.880; *p* < 0.001), further analyses were conducted only for the CASMIN index.

The outcome HLSAC differed significantly between questionnaire languages. Thus, regression analysis, controlling for German questionnaire language, urban residency, migration background, low and high FAS III, and low and medium CASMIN index was carried out. The corrected R^2^ = 0.019 was small but highly significant (*p* < 0.001). German questionnaire language (beta = 1.40 [0.62; 2.18], *p* < 0.001), low CASMIN index (beta = −1.51 [−2.27; −0.75], *p* < 0.001), and medium CASMIN index (−0.89 [−1.15; −0.30], *p* = 0.003) remained significant, while all other variables were no more significant.

### 3.4. Adolescents’ Mental Health Screening Outcomes and Their Associations to Proxy- and Self-Reported HL and Questionnaire Language

The correlations of metric adolescents’ mental health screening outcomes, GPIUS-2 scores, and MSPSS scores reported by parents and adolescents with parental HLS-EU-Q16 score, adolescents’ HLSAC scores, and questionnaire language are reported in Table 2. There was no significant correlation of mental health screening outcomes with German and Italian questionnaire language, despite the number of psychosomatic complaints in the adolescent reports. In the parent reports, MSPSS score and number of psychosomatic complaints were related to questionnaire language.

Higher self- and parent-reported mental health scores and higher GPIUS-2 scores were negatively related to parental and adolescent HL, while higher MSPSS scores were positively related to parental and adolescent HL. Relations of self-reported mental health scores were stronger with self-reported HLSAC than with the parent-reported HLS-EU-Q16 scores.

For scores correlating with questionnaire language, correlations with the sociodemographic variables migration background, urban residency, low FAS III, high FAS III, CASMIN low, and CASMIN medium were tested. Self-reported number of psychosomatic complaints was not related to any of these variables. Proxy-reported number of psychosomatic complaints was related to migration background (Point-biserial coefficient = 0.035 **), low CASMIN (0.039 **), and low FAS III (0.048 ***). Proxy-reported MSPSS score was related to urban residency (0.031 *), low CASMIN (−0.123 ***), low FAS III (−0.05 ***), and high FAS III (0.046 ***).

Regression analysis for parent-reported MSPSS controlling for questionnaire language and other significantly related variables returned a small corrected R^2^ = 0.016 (*p* < 0.001) and low CASMIN as a significant variable (beta = −0.57 [−0.69; −0.44], *p* < 0.001). Questionnaire language was no longer significant. Regression analysis for parent-reported number of psychosomatic complaints returned a significant (*p* < 0.001) R^2^ = 0.015 with significant variables low FAS III (beta = −0.131 [−0.24; −0.02], *p* = 0.023) and low CASMIN (beta = −0.574 [−0.70; −0.45], *p* < 0.001). Questionnaire language was no more significant after adjustment for other socioeconomic variables.

### 3.5. The Explorative Indirect Effect Models

Linear regression analyses for the main outcomes were performed. Explorative indirect effect modeling was performed to explore the effects between HL scores, mental health screening outcomes, and further predictors. Mental health screening outcomes were the dependent variables, HLS-EU-Q16 or HLSAC accounted for indirect effects, and directly associated variables were assumed to be MSPSS and GPIUS-2. The indirect effect must exhibit significant relations to both the predictors and the outcome

#### 3.5.1. Associated Variables

Regarding both HL measures, it was shown in Section 3.3 and Section 3.4 that the significant association statement holds for metric variables MSPSS and GPIUS-2. Both HL measures were related positively to all metric outcomes.

HLS-EU-Q16 score was associated significantly with the following nominal or ordinal variables: FAS III category, urban residency, parental age group and CASMIN index. HLSAC was related significantly to FAS III category, questionnaire language, and CASMIN index.

For indirect effect modeling, all variables must be related to HL scores as well to mental health screening outcomes. This holds for the following variables:

For self-reports, GPIUS-2 total score was related to self-reported SDQ total score (0.507; *p* < 0.001), PHQ-2 score (0.419; *p* < 0.001), SCARED score (0.405; *p* < 0.001), and self-reported number of psychosomatic complaints (0.402; *p* < 0.001).

Self-reported MSPSS score was related to SDQ score (−0.367; *p* < 0.001), PHQ-2 score (−0.306; *p* < 0.001), SCARED score (−0.251; *p* < 0.001), number of psychosomatic complaints (−0.290; *p* < 0.001), and GPIUS-2 score (−0.114; *p* < 0.001).

Parent-reported MSPSS score was related to parent-reported SDQ score (−0.259; *p* < 0.001) and parent-reported number of psychosomatic complaints (−0.172; *p* < 0.001). FAS III category was related to parent-reported SDQ score (−0.071; *p* < 0.001) and parent-reported number of psychosomatic complaints (−0.058; *p* = 0.003).

CASMIN index was related to parent-reported SDQ score (−0.080; *p* < 0.001). Residency was not related to any of the mental health screening outcomes. Parental age group was related to SCARED GAD-9 (0.058; *p* = 0.025), PHQ-2 (0.062; *p* = 0.026), and self-reported number of psychosomatic complaints (0.076; 0.004). Finally, as shown in Table 1, questionnaire language was related to number of psychosomatic complaints in self-reports (even in parent reports), but there was no relation to HLS-EU-Q16 score.

#### 3.5.2. Regression Analysis for HLS-EU-Q16 and HLSAC

Regression analysis controlling for confounders was performed for HLS-EU-Q16 score and HLSAC score. Results are presented in Table 3. For HLS-EU-Q16 higher MSPSS (*p* = 0.006) score was significantly related to higher HLS_EU-Q16 score as well as low (*p* = 0.017) and medium (*p* < 0.001) CASMIN index (CASMIN high used as baseline). The overall model (F = 6.606; *p* < 0.001) shows a corrected R^2^ = 0.017. All VIF-values were <1.2. Residuals centered around 0 but were slightly right skewed. Centered leverage point analyses returned some outliers, but recalculation of the models leaving them out confirmed the former results. Finally, we added self-reported GPIUS-2 score to the model. The variable was significant, with the former variables also remaining significant. The overall model (F = 6.523; *p* < 0.001) with corrected R^2^ = 0.042 remained stable and did not change. Results are not presented in Table 2 due to the length of the paper.

For HLSAC, higher MSPSS (*p* < 0.001), German questionnaire language (*p* = 0.003), higher children’s age (<0.001), and lower GPIUS-2 (<0.001) were associated with higher HLSAC score, while low (*p* < 0.001) and medium (0.026) CASMIN index were associated with lower HLSAC score. The overall model (F = 16.43; *p* < 0.001) showed a corrected R^2^ = 0.086; VIF was <1.3 for all independent variables. Residuals were centered around 0 with quite randomly distributed variance and the normal PP-Plot showed that residuals clustered around the line. Centered leverage point analyses returned some outliers. Recalculation of the models leaving them out confirmed the former results.

#### 3.5.3. Results of Indirect-Effect Modeling

For the strongest associates of mental health screening outcomes MSPSS and GPIUS-2, we applied model 4, modeling the associations of these two predictors with different mental health screening outcomes. We used HL as an indirect effect. Self-reported predictors were related to self-reported outcomes. The same holds for parent-reported data when possible. Parent-reported HL was used as an indirect effect for both parent- and self-reported data, while self-reported HL was used as an indirect effect for self-reported data.

Significant results of the models are presented in Table 4. For the outcome “self-reported SDQ”, the sample size was N = 1367, and the number of bootstrap samples was 5000. MSPSS (*p* < 0.001) and GPIUS-2 (*p* < 0.001) were significant direct associates. In both indirect effect paths, MSPSS (*p* < 0.001) and GIUS-2 (*p* < 0.001) were significantly associated with HLSAC, and HLSAC was significantly associated with SDQ (*p* < 0.001). Model coefficients for direct effects with confidence intervals and for indirect effects with bootstrap confidence intervals are presented in Table 4. HL accounted for about 10% of the total relationship between MSPSS and self-reported SDQ, and for about 6% of the relationship between GPIUS-2 and self-reported SDQ. Rerunning the model with 10,000 bootstrap samples showed stable confidence intervals. Modeling the same model using parental HLS-EU-Q16, the indirect effect was not significant, thus, the results are not reported.

For SCARED (GAD-9), the sample size was N = 1402, and the number of bootstrap samples was 5000; MSPSS (*p* < 0.001) and GPIUS-2 (*p* < 0.001) were directly related to the outcome. There was a significant indirect association represented by the associations of HLSAC with GPIUS (*p* < 0.001), MSPSS (*p* < 0.001), and the outcome (*p* = 0.02). Coefficients are presented in Table 4. HLSAC accounted for about 7% of the relationship between self-reported SCARED-GAD-9 scores and MSPSS and for about 3% of the relationship between self-reported SCARED-GAD-9. Rerunning the model with 10,000 bootstrap samples showed stable confidence intervals. Modeling the same model using parental HLS-EU-Q16, no significant indirect effect was found.

For self-reported PHQ-2 score, the relation between HLSAC and PHQ-2 score was not significant, and thus no indirect effect of HLSAC was detected. The same holds for HLS-EU-Q16.

The outcome self-reported “number of psychosomatic complaints” was directly related to MSPSS (*p* < 0.001) and GPIUS-2 (*p* < 0.001). The sample size was N = 1390, and the number of bootstrap samples was 5000. There was a significant indirect association represented by the associations of HLSAC with GPIUS-2 (*p* < 0.001), MSPSS (*p* < 0.001), and the outcome (*p* = 0.004). Coefficients are presented in Table 3. HLSAC accounted for about 6% of the relationship between self-reported number of psychosomatic complaints and MSPSS, and about 4% of the relationship between self-reported number of psychosomatic complaints and GPIUS-2. Rerunning the model with 10,000 bootstrap samples showed stable confidence intervals.

For parent-reported SDQ, Table 4 shows that the outcome was directly related to self-reported GPIUS-2 (*p* < 0.001) and parent-reported MSPSS (*p* < 0.001). The sample size was N = 1179, and the number of bootstrap samples was 5000. There was a significant indirect association represented by the associations of HLS-EU-Q16 with GPIUS-2 (*p* < 0.001), MSPSS (*p* = 0.007), and the outcome (*p* < 0.001). HLS-EU-Q16 accounted for about 10% of the relationship between parent-reported SDQ and parent-reported MSPSS and for about 6% of the relationship between parent-reported SDQ and self-reported GPIUS-2. Rerunning the model with 10,000 bootstrap samples showed stable confidence intervals.

For parent-reported “number of psychosomatic complaints”, Table 4 shows that the outcome was directly related to self-reported GPIUS-2 (*p* < 0.001) and parent-reported MSPSS (*p* < 0.001). The sample size was N = 1221, and the number of bootstrap samples was 5000. There was a significant indirect association represented by the association of HLS-EU-Q16 with GPIUS-2 (*p* < 0.001), MSPSS (*p* = 0.002), and the outcome (*p* < 0.001). HLS-EU-Q16 accounted for about 10% of the relationship between parent-reported number of psychosomatic complaints and parent-reported MSPSS, and for about 7% of the relationship between parent-reported number of psychosomatic complaints and self-reported GPIUS-2. Rerunning the model with 10,000 bootstrap samples showed stable confidence intervals.

Recalculation of the model using parent-reported MSPSS as a single indirect effect returned HLS-EU-Q16 to account for about 11% of the relationship between MSPSS and parent-reported SDQ, and about 11% of the relationship between parent-reported number of psychosomatic complaints and MSPSS.

### 3.6. Participants with Missing Parental HL Score

The distribution of categorized HL data, including missing HL datasets among parents completing the German questionnaire version, was as follows: 7.5% [6.7%; 8.4%] reported an inadequate HL, 18.3% [17.0%; 19.6%] a problematic HL, 53.8% [52.1%; 55.5%] an adequate HL, and for 20.4% [19.0%; 21.8%] some answers were missing. Among Italian parents, 6.3% [4.7%; 8.4%] reported an inadequate, 21.5% [18.5%; 24.7%] a problematic, and 59.8% [56.0%; 63.5%] an adequate HL, while 12.4% [10.0%; 15.1%] were coded as missing. The difference between the two questionnaire language groups was significant (Phi = 0.081; *p* < 0.001). Self-reported adolescents’ HL was low in 10.3% [8.7%; 12.0%] of the German questionnaires, middle in 63.8% [61.2%; 66.4%], and high in 23.1% [20.9%; 25.4%], with 2.8% [2.0%; 3.8%] missing. For Italian questionnaires, there were found 13.1% [9.2%; 17.8%] of low, 69.2% [63.1%; 74.8%] of middle, and 14.3% [10.3%; 19.2%] of high HL with 3.4% [1.6%; 6.3%] missing. The difference between the two questionnaire language groups was significant (Phi = 0.020; *p* = 0.021).

Finally, we were interested in whether the group of participants with more than two missing answers regarding parental HL (19.1%), resulting in a missing score, differed significantly from the group of parents with available HL score. Significant results were presented after Bonferroni correction for 13 tests (*p* < 0.05/13 = 0.00385 significant). Despite the differences between questionnaire languages, we found a significant higher percentage of missing cases in parents with lower CASMIN index (low: 26.6%; medium: 21.3%; high: 12.8%; *p* < 0.001), parental age group (21–39: 23.3%; 40–49: 20.1%; 50+: 16.5%; *p* = 0.003), and parents indicating a lower FAS III (low: 23.8%; medium: 19.2%; high: 15.4%; *p* < 0.001). No significant differences were found for single parenthood, migration background, and parental gender. Language specific differences are listed in Table 5. No Bonferroni correction was applied due to small sample sizes in the Italian sample. In both samples, participants with lower CASMIN index had significantly more than two missing answers more often. For the Italian sample, participants with migration background had more than two missing answers more often. Participants completing the German questionnaire version and having lower FAS III had more than two missing answers more often.

Regarding metric variables, significant results were presented after Bonferroni correction for 9 tests (*p* < 0.05/9 = 0.0056 significant). The two groups differed significantly for parent-reported MSPSS score with lower MSPSS score in the not missing group (effect size = 0.062; *p* < 0.001) and PHQ-2 score (effect size = 0.078; *p* = 0.002). No significant differences were found for self-reported MSPSS score, GPIUS-2 score, self- and parent-reported number of psychosomatic complaints, SCARED GAD-9 score, and self- and parent-reported SDQ total score.

## 4. Discussion

The study comprehensively examined parental and adolescent HL and its association with youths’ mental health screening outcomes in a bilingual region. The findings should be interpreted as evidence from a large school-recruited responding sample and may not fully represent all families and adolescents in the region.

Although the age distribution corresponds to the age distribution of the school-children population in South Tyrol, we cannot be sure about the representativeness of the data. Thus, the study can provide indications but may over- or underestimate the real situation. The first finding was that adolescents’ HL is related to survey language, with better results for the German questionnaire language. Parental HL was not related to survey language. Second, adolescent and parental HL were associated with adolescents’ mental health screening outcomes. Adolescent HL related more strongly to adolescents’ mental health screening outcomes than parental HL. Additionally, social support and problematic Internet use were related to both mental health screening outcomes and parent- and adolescent-reported HL. Exploratory cross-sectional indirect effect modeling found that self-reported HL partially accounted for the relation of self-reported mental health screening outcomes with MSPSS and GPIUS-2.

### 4.1. Parental and Adolescents’ HL

Parent-reported HLS-EU-Q16 and adolescent self-reported HLSAC are two different HL instruments and are not parallel forms of the same measure. They assess related but developmentally different self-reported competencies. Parent HL may reflect adult navigation of health information and services, while adolescent HLSAC reflects young people’s perceived capacity to access, understand, appraise, and apply health information in an age-appropriate context. Their interpretation, even if there exists a relation, is carried out independently.

In South Tyrol, parent-reported HL was found to be adequate in more than 50% of participating parents with a very high rate of not evaluable cases. A similar study in Germany [77] confirms our results, showing a similar rate of parents having an adequate HL. Positive relations between better HL and higher parental age as well as higher socioeconomic status, including educational level, confirm our results.

Generally, the data must be interpreted with caution. We conducted an anonymous online survey with email invitations, which yielded a response rate of approximately 23%. The value and practicability of online child mental health surveys were discussed in [78], showing that with a response rate of approximately 20%, the results mostly replicate the results from other studies.

Additionally, the large number of questionnaires that were not completed has not been found in similar studies outside the region, but has been found in former investigations in South Tyrol [79]. This may introduce bias, especially since the missing data correlate with lower parent-reported social support (MSPSS). This bias could lead to an underestimation or overestimation of HL’s indirect effect on adolescent mental health screening outcomes.

Despite the above-mentioned selection bias among parents, there may be further selection bias among adolescents. Nearly half of adolescents have not completed questionnaires in general, which we have previously discussed in [18]. There was no difference in socioeconomic characteristics, like low parental education, FAS III, migration background, or residency between the answering and the not answering group of adolescents but differed in proxy-reported MSPSS with better social support in the answering group. This effect is interesting, since it was even found in the missing parent-reported data. A first interpretation can be made on proxy-reported MSPSS: lower scores are associated with incomplete proxy- and adolescents’ questionnaires, thus parents and adolescents with a low social support were less likely to participate in the survey. Since lower MSPSS was one of the strongest correlates to lower parental and adolescents’ HL, we may assume that the overall HL rates in the population were lower. Thus, socially disadvantaged families can be identified as a vulnerable subgroup for the implementation of HL programs. School settings are a good environment to identify and support this group. Additionally, parents with a lower educational attainment were more likely to only partly complete the questionnaire. A general underestimation of low HL is probable. Thus, lower educated families, especially the parents, may be a target group in HL implementation planning. When implementing HL programs for parents, this fact may be considered. Lower educated parents may even be underrepresented in voluntary school-based HL programs. Special invitation strategies may be applied to foster the participation of this target group.

In Italy [6,80], adults’ HL was measured in a comparable sample regarding age and educational level. While only 33% had an adequate HLS-EU-Q16 score, about 60% had high HL when using the Newest Vital Sign (NVS-IT) questionnaire. Authors found that higher educational level, higher age class, and better financial resources were significantly associated with higher HL skills. Since our results showed a positive relation of parental HL to older age, higher FAS III, and higher CASMIN index, parent-reported HL in South Tyrol aligns with HL in other Italian studies.

Adolescents’ self-reported HL was found to be high in more than 20% of cases. A similar study in Italy in 2021 [10] found a corresponding rate of 6.8% and stated that the HL of Italian students was the lowest in Europe. At the same time, in Germany, the HL of adolescents and their parents was investigated. The study identified 14.2% of adolescents as having a high HL, using the same instrument [13]. The same study found about 60% of adults to have an adequate HL [81], and about 50% of the teachers [82]. These results align with our results. Thus, our results are placed in the European context and can relay on already existing school intervention programs in general when conducting intervention planning.

The HL of parents and adolescents has been measured using different instruments, instruments widely used in European and international contexts. Thus, interpretation of each instrument alone may be undermined by international evidence. Comparison of both instruments is not possible as they target different groups. Parental health literacy focuses more on general understanding and dealing with health information. Adolescent HL is embedded in a more developmental context, providing information on how adolescents integrate in their environment. Each instrument provides information about how participants act on their own health. There exist correlations between both measures, but the scores cannot be related directly to each other.

### 4.2. HL in a Bilingual Context

No significant difference in parental HL between questionnaire languages was found in fully completed questionnaires. The results of the study should be interpreted with caution when comparing former published research. First, the levels of HL using the Italian HLS-EU-Q16 tool were higher than those observed in former population-based research [34,83], while considering the NVS-IT, it was the opposite situation [84]. Furthermore, the authors pointed out differences not only between countries, but even between instruments within the same population.

The insight, that there is no difference between adults’ questionnaires in German and Italian questionnaire language in South Tyrol may be misleading. Completed parental HL was reported significantly more often in the Italian than in the German questionnaires. This is an interesting result, indicating that within the same healthcare system, the HLS-EU-Q16 questionnaire returns similar results and thus may be more easily comparable than that found in former research, where cultural differences between the Italian version and other European versions were discussed [7]. On the other hand, missing data differed in questionnaire language and parental educational status. This result is reflected in the CASMIN index disparities, since participants completing the German version more often had a lower educational status. Furthermore, missing HL was found more often in younger parents and participants with lower FAS III. While age was a questionnaire language independent factor, Italian participants were more likely to complete the questionnaire than German participants when having a lower FAS III. These results identify lower educated and lower income parents as a target group when promoting HL, especially when promoting the German questionnaire version.

Generally, adolescents’ HL was reported in both questionnaire languages nearly always completely. Lower adolescent HL in Italian questionnaires has been found, supporting the findings of [10]. When controlling HLSAC score for socioeconomic factors, questionnaire language remained a highly significant factor. Despite questionnaire language, low CASMIN index remained highly significant, and medium CASMIN index was also significant. Thus, these two factors need attention when implementing HL programs at school. Children from lower educated parents are a special target group, and the implementation of bilingual school-based interventions needs to be accompanied by language specific monitoring.

Higher scores in German HLSAC items regarding information finding behaviors, critical information comparison, and interpretation and self-awareness may be due to different wordings in the questionnaire. Since all items regarding health information were lower for Italian questionnaires, our results may align with the findings from [10]. The higher score of the German instrument may be explained by different digital information seeking and the fact that in Germany, there has already been a lot done to introduce specific HL programs for adolescents [85,86]. The availability of a broader range of health promotion resources for the German speaking adolescents may have contributed to a higher HL score in the German questionnaire version. Additionally educational differences may emerge from the South Tyrolean dual school system. German and Italian schools are managed independently from each other, so a common HL concept would be meaningful in light of future research.

The pattern of language differences between adolescents’ HL questionnaire versions was not found for parents. Cultural differences in health-related adult information seeking in South Tyrol have already been investigated in [1,87]. The authors showed that German speakers were more likely to rely on interpersonal and professional sources, while Italian speakers showed greater engagement with digital health sources. The observed differences may be due to cultural media consumption patterns, healthcare communication strategies, or varying levels of trust in public institutions across linguistic groups. Further sociocultural factors may explain differences in health information seeking behavior, since the German population lives predominantly in rural areas, while the Italian population lives in urban areas. Cultural differences in parental health information seeking behavior may not reflect adolescent behavior, since adolescents may be more engaged in Internet seeking strategies.

From these insights, it makes sense to develop targeted HL programs for parents and children in South Tyrol in both languages. Parental programs should address the importance of having a good HL independently from economic and educational background, while for adolescents, targeted school-based programs in both languages could be developed and tested in the bilingual context. The aim of the introduction of such programs may be to improve HL, and may especially work on existing differences between language groups. Longitudinal investigations may monitor the process, investigating whether differences between questionnaire languages persist over time after adjustment for sociodemographic associates. Since a wide range of German HL programs for adolescents already exist, a detailed study of these programs may be the basis of HL intervention planning in the Italian healthcare system and policy context. A special focus may be placed on information seeking and information interpretation as well as self-awareness. Providing an actual adapted bilingual program makes sense and can be a first step to improve HL in German and Italian adolescents in general.

Finally, language investigations provide evidence that parent-reported mental health scores are not influenced by language, since after controlling for sociodemographic parameters, any language relation disappears. Adolescents’ reports showed language differences in HLSAC and number of psychosomatic complaints, which did not disappear after correction for possible confounders. This finding is interesting in the context of general further healthcare and HL planning. While schools may provide targeted programs focusing on language differences between reported data, parental HL knowledge improvement may mainly address socioeconomic inequalities.

### 4.3. Demographic and Psychosocial Determinants

The large sample size of the dataset raises several questions. Effect sizes were small, but significant, thus the data must be interpreted with caution. Significant findings may relate to the large sample. On the other hand, significant associations of lower parental and adolescents’ HL with low FAS III, MSPSS, and low parental education have been demonstrated in former research [4,8,9,10,13]. Additionally, the high educational status of participating parents may have led to an underrepresentation of parents and adolescents with lower HL and socioeconomic resources. Importantly, incomplete HL responses were associated with lower educational and socioeconomic indicators. Thus, the data may systematically overestimate HL among parents. These insights raise the question of how HL may be spread best among the population. School-based HL programs are a promising tool to reach all young people, independent of their sociocultural backgrounds. At the same time, they offer the possibility to reach all parents. Inclusive strategies, providing information at an economic level among school settings and targeting whole families can serve to disseminate HL as an intergenerational concept. In Refs. [88,89], the authors discuss that schools can be seen as an ideal venue for strengthening HL because they reach almost all school-aged children throughout their school years. Integrating HL into schools is challenging, as both schools and teachers already face numerous educational requirements that may prevent them from addressing health in the classroom because they perceive it as an additional task. In this context, curriculum and instruction on media literacy, information literacy, and digital literacy are most promising subjects to include HL because these concepts share many commonalities with HL and are often already part of the school curriculum.

### 4.4. Adolescents’ Mental Health and HL

Adolescents’ and parental general HL was related to adolescents’ mental health screening outcomes. It was evident that higher self-reported mental health scores were stronger related to lower self-reported adolescents’ HL than to lower parent-reported HL. Higher parent-reported adolescents’ mental health scores were related to both lower parental and adolescents’ HL. Adolescents’ HL may have a stronger association with adolescents’ mental health outcomes, because it acts on the individual and proximal level, shaping self-regulation, coping, and decision-making. Parental health literacy affects mental health outcomes more indirectly via family processes (e.g., communication and support) [90].

Future work should test whether mental-health-specific literacy or general school-based HL interventions affect the mental health screening outcomes of adolescents over time. Strengthening the HL of both parents and children may make sense to prevent and early detect health problems and psychosocial problems in young people.

Higher problematic Internet use was related to mental health screening outcomes. The relation may be bidirectional. Indirect effect models confirmed the association with self-reported adolescents’ HL. Future investigations can focus on this relation, and on how HL may act in both directions. First, HL may act as a protective factor by enabling adolescents to deal with online information. Similarly, insufficient HL may increase misinterpretation, maladaptive information-seeking behavior, and uncertainty [43]. Conversely, problematic Internet use may affect HL by impacting information processing and cognitive functioning by information overload and misperceptions [91].

Parental HL was not a significant indirect effect in self-reported mental health screening outcomes. This result emphasizes the importance of respecting and prompting adolescents’ autonomy in health behavior. It aligns with the smaller correlation coefficient between parental HL and self-reported adolescents’ mental health screening outcomes. Factors like MSPSS and problematic Internet use interact with mental health screening outcomes independently of parental HL. Thus, when implementing school-based HL programs, it is important to preliminarily address problematic Internet use and thus digital HL in adolescent programs. The HL of socially lower supported adolescents should be strengthened so that adolescents are able to help themselves alone. The main target may be to improve the HL of children and adolescents. Improving the HL of parents and teachers is also important. First, they can help young people to improve their own HL. In [92], a tool for teaching critical HL to secondary school students was developed and found to be feasible and likely to enhance the competence of critical HL.

Second, good adult HL knowledge is needed even in early detection and dealing with the health problems of children and adolescents. In Ref. [82], more than half of the teachers showed a limited level of HL, and an association between low level of HL and uncertainty in dealing with chronically ill pupils was found. To prevent such uncertainties, it makes sense to generally improve the parents and teachers’ HL.

Theoretical frameworks to implement comprehensive school-based intervention concepts have been discussed in [88,89,93] to provide standards for the development of organizational HL in schools.

Despite all of these findings, there remains uncertainty regarding cross-sectional data and the fact that higher educated parents, especially mothers, completed the questionnaire. Lower health literacy in parents may be underestimated, and thus relationships between self-reported HL outcomes of adolescents with parental HL may have exhibited other patterns when investigating representative data.

### 4.5. Methodological Considerations

South Tyrol’s bilingual context offers a unique opportunity to adapt and implement HL programs across German and Italian schools. Sophisticated targeted HL programs are needed, addressing disparities in parental educational attainment, different social support, lower socioeconomic status and language related disparities in information finding and interpretation. Parental HL programs may target general HL, especially for disadvantaged groups. The aim may be to provide good HL for all parents to manage and early detect children’s health problems. Adolescent HL may address information appraisal, self-regulation, and autonomy in self-awareness and in dealing with digital media in both languages. Lower socially supported groups may be targeted. To obtain further evidence, long-term investigations may provide evidence about the possible effects of improved HL on young people’s mental health. Longitudinal monitoring may provide indications about the development of any non-questionnaire language-related difference.

Notably, HL in Germany worsened between 2014 and 2020 [94]. This effect was particularly evident among people with low social status and financial deprivation. The HL of the German population tended to improve during the pandemic [95]. Women, people with low or medium education, younger people, and those with a migration background appeared to have benefited to a more than average extent. To surmount socioeconomic differences in HL development in the younger populations, strategies to reach the whole population in the same way are needed.

For the future planning of HL developmental programs for adolescents, we can address schools as an important partner: Schools can reach all young people and their parents at the same time. Socially disadvantaged as well as socially advantaged parents and adolescents are reached, and no costs should emerge for families when HL interventions are applied. Thus, parents and adolescents from all socioeconomic backgrounds have the same possibilities to develop an adequate HL. The parallel involvement of teachers, parents, and adolescents is needed to provide a comprehensive program. The collaboration of healthcare institutions and schools is essential. A scientific longitudinal guidance may incorporate assessing and developing teachers’ HL empowerment and the intervention of school nurses providing HL information to children, adolescents, and parents. Such programs in Italy are lacking.

Between 2005 and 2015, HL was promoted and discussed widely in Germany. Starting with the main goal [96] to strengthen the scientific basis for decision-making in German healthcare services, promotion of general health- and scientific knowledge (critical health information, or HL) is strongly associated with good health outcomes and patient empowerment. Autonomy, empowerment, and HL were regarded as useful theoretical concepts to guide the definition of evidence-based health information on websites [74]. School nurses [97] providing health related expertise increased the HL of children, parents, and teachers.

The findings support the value of developing and prospectively evaluating bilingual school-based health literacy programs in South Tyrol, with attention to questionnaire-language group differences and socioeconomic inequalities. Its implementation in the educational environment makes sense since it offers the possibility to reach all young people independent from their sociocultural background. Strengthening the HL knowledge of parents by especially addressing German speaking low educated persons and improving teachers’ HL regarding adolescents’ mental health and general health should also be included in the concept.

### 4.6. Strength and Limitations

This study has several strengths. It involved parental and adolescent HL skills at once and associated them with both parent and adolescent-reported mental health screening outcomes. The whole study finds place in a bilingual context and offers the possibility of bringing together knowledge of both realities and developing new concepts adapted for this special region, but applicable in a broader context, especially in Italy.

Research is limited by the fact that the study is cross sectional and does not provide longitudinal data. Results remain explorative and no directional or causal interpretation could be carried out. Cross sectional indirect effect modeling in this context was used as an explorative tool, providing information about associations. Only longitudinal observations can provide evidence about the mediating effects of HL. Furthermore, the study only asked for general HL and not especially about mental or digital HL. Even if the aim was to exactly provide information about HL, future work may analyze HL as well as mental and digital HL in the context of adolescent mental health screening data. The Italian participation rate was low, which may have caused a response or selection bias. The use of self-report instruments, even if used from a dual perspective, may introduce bias or cause response biases like social desirability bias. Selection bias may be due to the high rates of female and higher educated parental participants. Finally, a high drop out in parental reported HL may under- or overestimate the indirect effect of parental HL, especially given the fact that in both language groups, lower parental education status was not only related to lower HL, but even to lower response rates, which needs attention in interpretation. Parental HL may have been overestimated with missing information for lower educated groups. Further research is needed to understand whether questionnaire language is a proxy for broader sociocultural factors. The small effect sizes of many sociocultural factors raise the question of whether significances are due to high sample sizes rather than due to real differences.

Future research directions may include the following steps: development of a bilingual HL intervention for South Tyrolean schools including tools for parents and adolescents. A longitudinal study may frame the test and introduction process. A special focus may consider sociodemographic and lingual disparities, especially parental educational status.

## 5. Conclusions

Our study provides evidence on the differences in adolescent HL between questionnaire languages in a bilingual region. It confirmed associations with different socioeconomic backgrounds and showed the associations between parental and adolescent HL and adolescents’ mental health. School-based interventions in German and Italian settings are needed, targeting teachers, parents, and students. Germany’s HL programs may act as underlying information but must be adapted to the bilingual situation and the Italian healthcare system.

Examination of teachers’ HL may be the next step in developing targeted intervention programs. The combined information on the parental, adolescent, and teachers’ actual HL in combination with sociocultural effects can be the basis for adapting existing HL programs to special regional needs.

Longitudinal research examining HL knowledge of all three target groups in the intervention program can provide future insights into the development of the South Tyrolean HL intervention program.

Additionally, it can be investigated whether targeted mental HL programs for adolescents or general HL programs are related to adolescents’ mental health. Parents may obtain information about prevention and the early detection of mental health conditions and about pathways in the healthcare system to deal with such problems.

## Figures and Tables

**Table 1:** Item level-descriptive comparison between German and Italian questionnaire language.

Items	Questionnaire Language	N	Mean + −SD	Median [25%; 75%]	*p*-Value GER vs. IT	Not at All (%)	Not Quite (%)	Somewhat (%)	Absolutely (%)
Having good information regarding health (1)	GER	1087	3.25 + −0.66	3.00	n.s.	1.4%	8.3%	53.6%	36.7%
	IT	215	3.25 + −0.59	3.00		1.4%	4.2%	63.3%	31.2%
Ability to give examples of things that promote health (2)	GER	1078	3.01 + −0.77	3.00	n.s.	4.2%	16.5%	53.4%	25.9%
	IT	215	2.97 + −0.69	3.00		2.8%	18.1%	60.0%	19.1%
Ability to find health-related information that is easy to understand (3)	GER	1085	3.03 + −0.80	3.00	0.001	4.6%	16.7%	50.2%	28.5%
	IT	214	2.88 + −0.65	3.00		2.3%	22.0%	62.1%	13.6%
Ability to follow instructions given by doctors and nurses (4)	GER	1086	3.39 + −0.71	4.00	n.s.	1.5%	8.7%	38.7%	51.2%
	IT	214	3.34 + −0.62	3.00		0.9%	6.1%	51.9%	41.1%
Ability to decide if health-related information is right of wrong (5)	GER	1087	3.31 + −0.71	3.00	<0.001	1.4%	10.4%	43.6%	44.6%
	IT	213	2.97 + −0.73	3.00		2.8%	19.2%	55.4%	22.5%
Ability to compare health-related information from different sources (6)	GER	1086	3.30 + −0.67	3.00	<0.001	1.4%	8.2%	50.3%	40.1%
	IT	214	3.03 + −0.71	3.00		1.9%	19.2%	53.3%	25.7%
Ability to justify one’s own choice regarding health (7)	GER	1083	3.20 + −0.72	3.00	<0.001	1.7%	12.5%	49.5%	36.4%
	IT	214	3.00 + −0.71	3.00		3.7%	15.4%	59.3%	21.5%
Ability to judge how one’s own behavior affects one’s health (8)	GER	1083	3.30 + −0.66	3.00	<0.001	1.2%	7.7%	50.2%	40.9%
	IT	214	3.12 + −0.68	3.00		1.9%	12.6%	57.9%	27.6%
Ability to judge how one’s own action affects the surrounding natural environment (9)	GER	1083	3.03 + −0.72	3.00	n.s.	2.3%	17.6%	55.2%	24.8%
	IT	214	3.04 + −0.68	3.00		2.3%	15.0%	60.3%	22.4%
Ability to give ideas on how to improve one’s immediate surroundings (10)	GER	1083	3.19 + −0.71	3.00	<0.001	1.9%	11.7%	51.9%	34.4%
	IT	214	3.01 + −0.69	3.00		1.9%	19.2%	56.5%	22.4%

**Table 2:** Correlations of adolescents’ mental health scores with HLS-EU-Q16, HLSAC, and questionnaire language.

**Self-Reports**	**HLS-EU-Q16**		**HLSAC**		**Questionnaire Language (German = 1; Italian = 0)**	
	**Spearman**	***p*-Value**	**Spearman**	***p*-Value**	**Point-Biserial**	***p*-Value**
SDQ (N-G:1051; N-I:212)	−0.144	<0.001	−0.291	<0.001		n.s.
PHQ-2 (N-G:1096; N-I:215)	−0.126	<0.001	−0.157	<0.001		n.s.
SCARED (N-G:1075; N-I:215)	−0.107	<0.001	−0.173	<0.001		n.s.
Number of psychosomatic complaints (N-G:1082; N-I:217)	−0.120	<0.001	−0.177	<0.001	−0.078	0.002
MSPSS (N-G:1162; N-I:229)	0.115	<0.001	0.237	<0.001		n.s.
GPIUS-2 (N-G:1080; N-I:213)	−0.155	<0.001	−0.234	<0.001		n.s.
**Parent Reports**	**HLS Score**		**HLSAC Score**		**Questionnaire Language (German = 1; Italian = 0)**	
	**Spearman**	***p*-Value**	**Spearman**	***p*-Value**	**Point-Biserial**	***p*-Value**
SDQ (N-G:2183; N-I:472)	−0.221	<0.001	−0.275	<0.001		n.s.
Number of psychosomatic complaints (N-G:2210; N-I:491)	−0.200	<0.001	−0.158	<0.001	−0.061	<0.001
MSPSS (N-G:2437; N-I:529)	0.170	<0.001	0.159	<0.001	−0.038	0.024

N-G: Sample size German questionnaire language group; N-I: Sample size Italian questionnaire language group.

**Table 3:** Coefficients and standardized coefficients of linear regression analysis for HLS-EU-Q16 score and HLSAC score as dependent variables.

	Variables	Beta	Standardized Beta	*p*-Value
HLS-EU-Q16	Constant	13.27 [12.53; 14.02]		<0.001
	CASMIN low	−0.30 [−0.55; −0.05]	−0.051	0.017
	CASMIN medium	−0.42 [−0.60; −0.24]	−0.098	<0.001
	FAS low	−0.18 [−0.43; 0.07]	−0.029	0.161
	FAS high	0.13 [−0.05; 0.31]	0.028	0.168
	MSPSS	0.06 [0.02; 0.11]	0.054	0.006
	Single parenthood	−0.04 [−0.30; 0.22]	−0.006	0.767
	Parental age	0.01 [−0.01; 0.02]	0.025	0.2
	Residency	0.13 [−0.05; 0.30]	0.028	0.162
HLSAC	Constant	27.70 [25.53; 29.88]		<0.001
	CASMIN low	−1.60 [−2.35; −0.85]	−0.12	<0.001
	CASMIN medium	−0.66 [−1.23; −0.08]	−0.06	0.026
	FAS III low	−0.22 [−0.96; 0.52]	−0.02	0.558
	FAS III high	0.17 [−0.43; 0.78]	0.016	0.573
	GPIUS-2 score	−0.06 [−0.07; −0.04]	−0.222	<0.001
	Age	0.26 [0.15; 0.38]	0.122	<0.001
	German questionnaires	1.11 [0.39; 1.83]	0.08	0.003
	MSPSS score	0.37 [0.18; 0.55]	0.102	<0.001

**Table 4:** Indirect effect model 4 with indirect effect M = HLS-EU-Q16 for parent-reported and M = HLSAC for self-reported mental health scores, Y = SDQ (self + parent)/SCARED/PHQ-2/psychosomatic complaints (self + parent), with two independent predictors X1 = MSPSS score and X2 = GPIUS-2 score.

**SELF**	**c1′**	**c2′**	**c1**	**c2**	a1*b	**%**	a2*b	**%**
y = SDQ	−0.697 [−0.872; −0.521]	0.133 [0.120; 0.146]	−0.770 [−0.947; −0.594]	0.143 [0.130; 0.156]	−0.074 [−0.115; −0.039]	10	0.009 [0.006; 0.013]	6
y = SCARED	−0.301 [−0.463; −0.140]	0.088 [0.076; 0.100]	−0.325 [−0.486; −0.165]	0.091 [0.079; 0.103]	−0.024	7	0.003 [0.001; 0.006] [−0.050; −0.004]	3
y = npc *	−0.230 [−0.312; −0.148]	0.041 [0.035; 0.005]	−0.245 [−0.327; −0.164]	0.043 [0.037; 0.049]	−0.015 [−0.029; −0.005]	6	0.002	4
**PARENT**	**c1′**	**c2′**	**c1**	**c2**	a1*b	**%**	a2*b	**%**
y = SDQ	−0.404 [−0.574; −0.233]	0.099 [0.084; 0.114]	−0.450 [−0.622; −0.278]	0.105 [0.090; 0.120]	−0.046 [−0.087; −0.017]	10	0.006	6
y = npc *	−0.145 [−0.213; −0.078]	0.025 [0.020; 0.031]	−0.161 [−0.229; −0.094]	0.027 [0.021; 0.033]	−0.016 [−0.030; −0.006]	10	0.002 [0.001; 0.003]	7

* Number of psychosomatic complaints; c1′, c2′ direct effects, c1, c2 total effects, a1*b, a2*b indirect effects.

**Table 5:** Language specific differences between complete and not complete (more than two missing answers) HLS-EU-Q16 questionnaires.

		German		Italian	
		%	*p*-Value	%	*p*-Value
Gender	Female	19.7%	n.s.	10.7%	n.s.
	Male	21.0%		14.3%	
Single parenthood	Yes	21.5%	n.s.	10.6%	n.s.
	No	20.2%		12.7%	
Residency	Urban	19.4%	n.s.	13.2%	n.s.
	Rural	20.7%		10.3%	
Migration background	Yes	19.4%	n.s.	18.8%	0.024
	No	20.3%		10.8%	
CASMIN	Low	27.5%	<0.001	18.4%	0.048
	Medium	22.7%		13.7%	
	High	13.7%		9.2%	
FAS III	Low	26.5%	<0.001	13.1%	n.s.
	Medium	20.6%		11.1%	
	High	15.7%		14.3%	

## Data Availability

The datasets generated and analyzed during the current study are available from the corresponding author upon reasonable request.

## Abbreviations

The following abbreviations are used in this manuscript:

CASMIN	Comparative Analysis of Social Mobility in Industrial Nations
COP-S	Corona and Psyche South Tyrol
COPSY	Corona and Psyche
FAS	Family Affluence Scale
GPIUS	Generalized Problematic Internet Use Scale
HBSC-SCL	Health Behavior in School-Aged Children-Symptom Checklist
HL	Health Literacy
HLS-EU-Q	European Health Literacy Survey Questionnaire
HLSAC	Health Literacy for School-Aged Children
M	Mean
MSPSS	Multidimensional Scale of Perceived Support
n.s.	Not Significant
NSV-IT	Newest Vital Sign-Italian version
PHQ	Patient Health Questionnaire
SCARED-GAD	Screen for Child Anxiety Related Disorders-Generalized Anxiety Disorder
SD	Standard Deviation
SDQ	Strengths and Difficulties Questionnaire
VIF	Variance Inflation Factor
